# Assessment of vascular stiffness using different modalities in patients with systemic lupus erythematosus: a case control study

**DOI:** 10.1186/s43044-020-00062-4

**Published:** 2020-05-18

**Authors:** Waleed Ammar, Moataz Taha, Essam Baligh, Dina Osama

**Affiliations:** Department of Cardiology, Kasr Al Aini Hospital, Faculty of Medicine, Cairo University, Cairo, 11562 Egypt

**Keywords:** Systemic lupus erythematosus, Arterial stiffness, Flow-mediated dilatation

## Abstract

**Background:**

Cardiovascular disease is a major cause of morbidity and mortality in systemic lupus erythematosus (SLE) patients. Accurate risk stratification would require a simple, non-invasive index integrating all traditional and emerging risk factors. Vascular stiffness fulfills these requirements and has better predictive value for cardiovascular events than traditional risk factors in hypertensives and patients with coronary artery disease. Our aim was to determine whether arterial stiffness is increased in SLE patients compared to healthy controls and to correlate the arterial stiffness in SLE patients with cardiovascular risk factors, namely, hypertension and diabetes mellitus.

**Results:**

This study included 50 SLE patients and 50 age- and gender-matched healthy individuals. SLE patients had higher median aortic stiffness index (SI) and lower strain and distensibility, compared to controls (*p* value for all < 0.001). SLE patients had significantly impaired flow-mediated dilation (FMD) compared to controls: the median (range) in SLE patients was 8.82 (2.5–21.87), compared to 19 (12–37.5) in controls (*z* = − 7.695, *p* ˂ 0.001). Regarding quality arterial stiffness (QAS) parameters, SLE patients had significantly lower median carotid distension, distensibility coefficient, and compliance coefficient, with higher median carotid SI, carotid pulse wave velocity (PWV), and augmentation index (AI), compared to controls (*p* value for all ≤ 0.001). SLE patients had a higher median cf-PWV 6.5 m/s (4.8–11.8), compared to a median of 4.6 m/s (3.8–6.9) in controls (*z* = − 8.193, *p* ˂ 0.001). Linear regression analysis to adjust for hypertension and diabetes mellitus yielded a statistically significant difference between both groups for all of the above parameters (*p* = 0.014 for maximum carotid intima media thickness (IMT) and < 0.001 for remaining parameters), with the exception of the maximum carotid augmentation index (*p* = 0.184).

**Conclusion:**

SLE patients have significantly increased arterial stiffness and impaired FMD compared to healthy controls. This is true even after adjusting for hypertension and diabetes mellitus, highlighting the fact that SLE could be an independent cardiovascular risk factor. These findings emphasize the need for early management of SLE together with aggressive risk factor modification.

## Background

Cardiovascular disease is a major cause of morbidity and mortality in systemic lupus erythematosus (SLE) patients [1]. These patients have a higher incidence and an earlier age of onset of ischemic heart disease, carotid atherosclerosis, cerebrovascular stroke, and peripheral vascular disease, despite being mostly pre-menopausal females [1–3]. Moreover, cardiovascular mortality in patients with SLE has not improved over time [4]. SLE activity and disease duration increased the risk of vascular events in some studies [5]. Traditional cardiovascular risk factors, including hypertension, diabetes mellitus, dyslipidemia, and physical inactivity, only account partially for the elevated vascular risk in SLE patients [6, 7]. Vascular stiffness proven to have better predictive value for fatal and non-fatal cardiovascular events than traditional risk factors in hypertensives and patients with end-stage renal disease or coronary artery disease [8]. Arterial compliance, distensibility, and elasticity are all different aspects of arterial stiffness. Stiffness can be determined by measuring pulse wave velocity (PWV) in the aorta using a mechanotransducer, tonometer, echotracking, or Doppler probes or the superficial arteries (common carotid, common femoral, brachial, and radial arteries) using video-image analysis or echotracking devices. Finally, arterial stiffness can be assessed by measuring the augmentation index, which represents the augmentation of central pulse pressure during late systole by the earlier return of wave reflection due to arterial stiffening [9, 10].

A healthy endothelium maintains arterial elasticity, mainly through the production of nitric oxide. Endothelial dysfunction represents the initial step of atherosclerosis and correlates with arterial stiffness. The identification of elevated vascular risk in SLE patients may warrant aggressive use of antihypertensives, statins, and immunomodulating agents despite the lack of prospective studies that prove the value of this approach. Therefore, assessment of arterial stiffness can be useful to guide therapeutic decisions in these patients in the future.

## Aim of the work

To determine whether arterial stiffness is increased in SLE patients compared to healthy controls and to correlate the arterial stiffness in SLE patients with cardiovascular risk factors, namely, hypertension and diabetes mellitus.

## Methods

### Subjects

Patients were recruited from rheumatology department. Fifty patients fulfilled the Systemic Lupus International Collaborating Clinics (SLICC)/revised American College of Rheumatology (ACR) classification criteria (group A) [11]. Fifty age- and gender-matched healthy individuals were recruited as control group (group B). All subjects gave written informed consent to take part in this study. Cardiovascular assessment was conducted at the cardiology department. All subjects had a complete history and physical examination and laboratory work up as needed.

### Assessment of aortic stiffness

Using Esaote MyLab 60 (phased array sector probe PA240, frequency range 1–4 MHz) and Philips Envisor (phased array sector probe S4, frequency range 2–4 MHz). Calculation of aortic elasticity indices using M-mode transthoracic echocardiography (TTE) was done. The diameter of the ascending aorta was measured in the parasternal long axis view by 2D guided M-mode tracing. Measurements were performed 3 cm distal to the aortic valve. The systolic diameter was measured at the maximal anterior motion of the aortic valve, whereas the diastolic diameter was measured at the peak of the QRS complex on the simultaneously recorded electrocardiogram. The average of three consecutive measurements was calculated. The formulas used in the calculation of elasticity indices were as follows:

- Aortic β stiffness index = ln (SBP/DBP)/[(SD − DD)/DD], where ln = natural logarithm, SBP = systolic blood pressure, DBP = diastolic blood pressure, SD = systolic diameter, and DD = diastolic diameter [12]
- Aortic strain (%) = (SD − DD)/DD [13]
- Aortic distensibility (10^−3^ mmHg^−1^) = 2 × (SD − DD)/[(SBP − DBP) × DD] × 1000 [12]

### Assessment of endothelial function: flow-mediated dilation (FMD)

A linear array transducer LA523 (frequency range 5–12 MHz) of Esaote MyLab 60 machine was used to assess FMD as described before [14].

### Carotid artery intima media thickness (IMT)

Using the linear array transducer LA523 of Esaote MyLab 60 machine, the carotid intima media thickness (IMT) measurement was performed in the proximal part of the common carotid artery, 1 cm proximal to the carotid bulb as the maximum distance between the intima-lumen and adventitia-media interfaces in areas without carotid plaque. By using B-mode, color, and pulsed Doppler, the presence of athermanous plaques, their sites, number, and percentage diameter reduction was determined.

### Assessment of carotid stiffness parameters

**Fig. 1:**
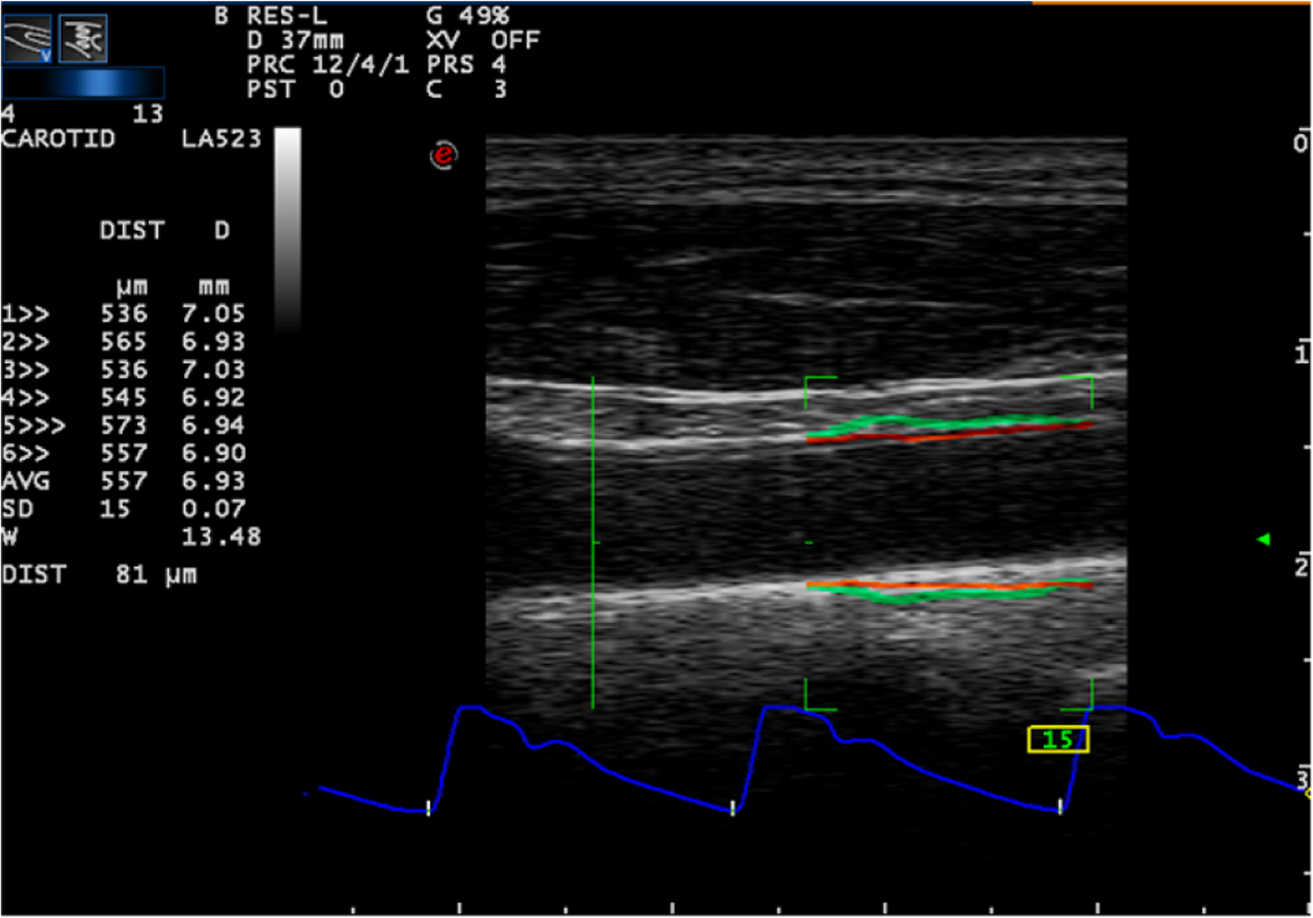
Quality arterial stiffness—radiofrequency wall-tracking system. The movement of carotid walls is tracked in the entire region of interest (green rectangle) composed of 32 scanning lines. Continuous orange lines indicate the automatic positioning of wall-tracking points at media-adventitia interface. Continuous green lines display dynamically the amplified vessel wall movement (the real vessel wall movement is “amplified” by a predefined factor). Real-time distension waveforms are displayed at the bottom (blue line). The values of carotid distension (DIST) and minimum diameter (D) are displayed beat-to-beat on the screen, and the mean value (MED) over the last six beats and standard deviation (SD) are continuously calculated. This results in a frame rate of 500 Hz that allows the detection of wall velocity distension up to 36 mm/s. Diameter wall tracking: continuous orange lines without interruptions indicate a good detection

Automated measurements were performed using QAS (quality arterial stiffness) software—a radiofrequency (RF) wall-tracking system. The vessel wall stiffness is expressed as pulse wave velocity obtained from brachial blood pressure and the accurate automated measurements of the vessel diameter and change in the diameter (Fig. 1). RF-based wall-tracking systems are capable of tracking arterial wall movement with adequate spatial and temporal resolution, as well as providing carotid pressure estimate from calibrated distension waveforms [15]. QAS provides a list of standard automatically calculated parameters combining the ultrasound-measured values (distension, distension waveform, and diameter) with the brachial blood pressure as follows:

1. *Distension* (DIST): the difference between systolic and diastolic diameter
2. *Compliance Coefficient* (CC): absolute change in vessel diameter (Δ*d*) during systole for a given pressure change (Δ*P*) [16]
3. *Distensibility Coefficient* (DC): relative change in vessel diameter during systole for a given pressure change [17]
4. Pulse wave velocity (PWV): the Bramwell-Hill equation relates the PWV to the distensibility coefficient (DC) [18]
5. Carotid β-stiffness index (SI): automatically calculated by the following equation: ln (SBP/DBP) X D/DIST [19]
6. Augmentation index (AIx) is calculated as the difference between the second and the first systolic peaks observed on the arterial waveforms, and it is expressed as a percentage of the pulse pressure
  AIx = [AP/(LocPsys − LocPdia)] × 100, where LocPsys is the local pressure, systolic; LocPdia is the local pressure, diastolic; and AP is the augmented pressure
7. Local blood pressure (Local SBP and DBP)

The central pressure waveform was determined non-invasively using diameter waveforms to derive pressure waveforms by appropriate calibration [20]. For each SLE patient/control, we reported the minimal (of both sides) DIST, DC, and CC and the maximal (of both sides) SI, PWV, and AI.

### Carotid femoral pulse wave velocity (cf-PWV)

**Fig. 2:**
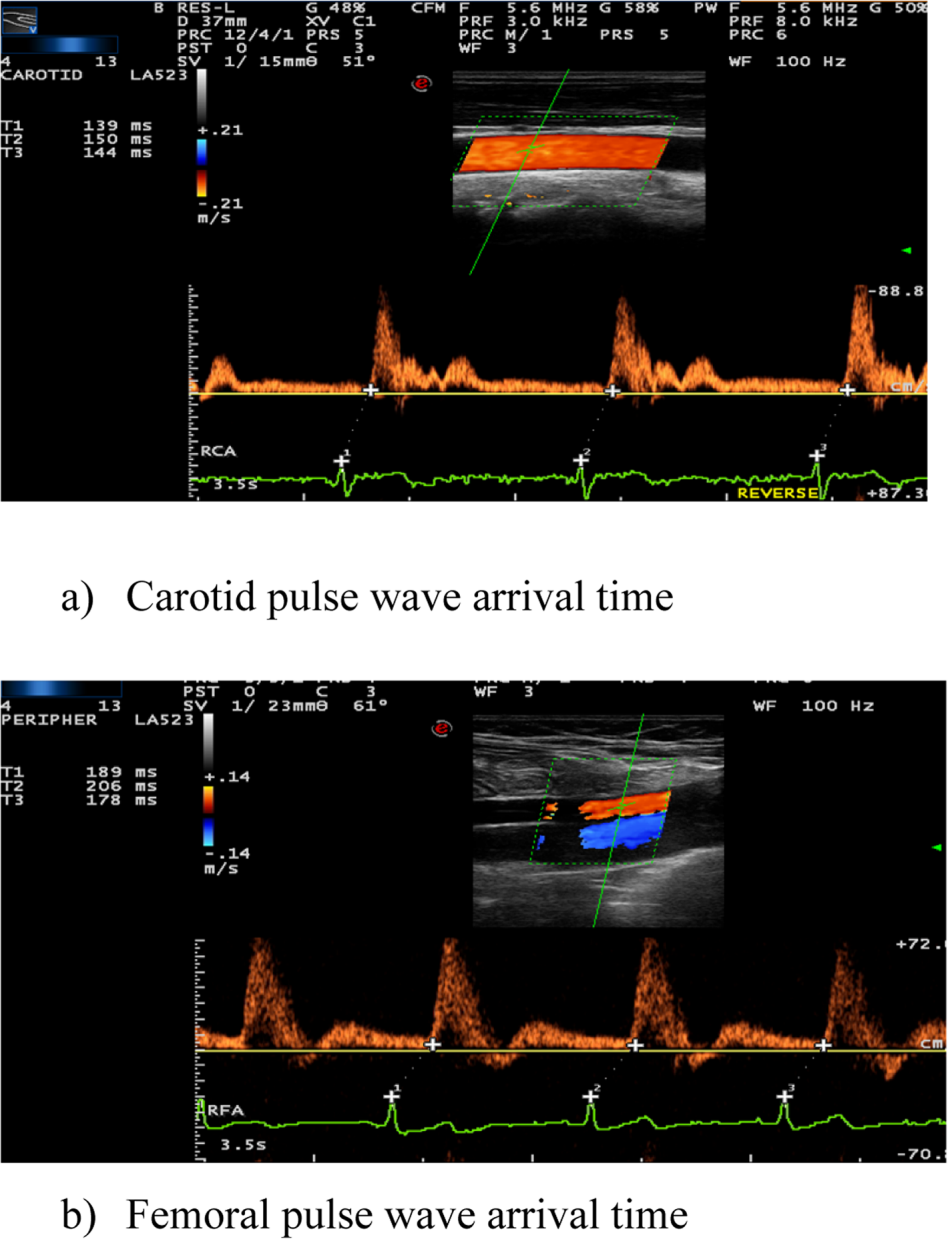
The time difference (Δ*t*) of pulse wave arrival at the carotid (**a**) and femoral (**b**) arteries, respectively [(F1 + F2 + F3)/3) − (C1 + C2 + C3)/3)]

This was calculated as the carotid-femoral travel distance divided by the transit time (Δ*L*/Δ*t*). The direct distance between the carotid and femoral measurement sites was used as Δ*L* [21]. To calculate Δ*t*, we subtracted the carotid from the femoral pulse wave arrival time. We took the average of 3 measurements for each of the carotid and femoral times; each was measured from the peak of QRS complex to the foot of the pulse wave (Fig. 2). The maximal cf-PWV of both sides was recorded for each patient/control.

### Statistical analysis

The distribution of data was studied with the Kolmogorov-Smirnov and Shapiro-Wilk tests. All data collected and analyzed in the study were non-normally distributed. The statistical analysis was performed using the SPSS version 23 statistical software. *p* values < 0.05 were considered significant for all analyses. Continuous variables were expressed as median (range) and discrete variables as percentages. Mann-Whitney *U* test was used to compare medians between SLE patients and controls regarding arterial stiffness parameters, FMD, and carotid IMT. Linear regression analysis was used to adjust for the effects of hypertension and diabetes mellitus. Regarding the predictors of arterial stiffness parameters in SLE patients, we used Mann-Whitney *U* test for comparison of stiffness parameters between the 2 categories of each qualitative variable. Bivariate correlation was used for quantitative variables. We assessed inter-observer variability for measurements of carotid stiffness and cf-PWV by calculating the intra-class correlation coefficient for a random sample of 5 SLE patients and 5 controls.

## Results

We studied 50 SLE patients and 50 healthy control subjects. In the patient group, Forty-seven patients (94%) were females; the median age of the patients was 29, range 18–45 years. The median SLE duration was 8 years (range 2–21 years). Twenty-three patients (46%) were hypertensives, and 8 patients (16%) were diabetics. Forty-five patients (90%) had renal impairment, five patients (10%) had history of stroke, and two (4%) had history of TIA.

### Aortic stiffness parameters

Assessment of aortic stiffness parameters using transthoracic echocardiographic M-mode analysis revealed a statistically significant difference between both groups as shown in Table 1.

**Table 1:** Aortic stiffness parameters in SLE patients (group A) and controls (group B)

Variable	Group A, median (range)	Group B, median (range)	*z*	*p*
Aortic SI	5.28 (1.79–30.45)	2.30 (0.75–6.82)	− 6.218	˂ 0.001
Aortic strain (%)	7.87 (1.77–20.8)	15.33 (6.45–35.17)	− 6.260	˂ 0.001
Aortic distensibility (10^−3^ mmHg^−1^)	3.47 (0.71–12.37)	8.95 (3.31–26.87)	− 6.852	˂ 0.001

*SI* stiffness index

SLE patients (group A) had higher aortic stiffness manifested by higher median stiffness index (SI) and lower strain and distensibility, compared to controls (group B).

### Endothelial function

SLE patients (group A) had significantly impaired endothelial flow-mediated dilation (FMD) compared to controls (group B): the median (range) in SLE patients was 8.82% (2.5–21.87), compared to 19% (12–37.5) in controls (*z* = − 7.695, *p* ˂ 0.001).

### Carotid intima media thickness (IMT)

There was a statistically significant difference in median carotid IMT between both groups. The median (range) IMT in SLE patients (group A) was 0.56 cm (0.35–1.1), compared to 0.49 cm (0.37–0.66) in controls (group B) (*z* = − 3.214, *p* ˂ 0.001).

### Quality arterial stiffness parameters (QAS)

SLE patients had significantly increased carotid stiffness compared to control group regarding the medians of all QAS parameters as shown in Table 2. Compared to controls (group B), SLE patients (group A) had significantly lower median carotid distension, distensibility coefficient, and compliance coefficient, with higher median carotid SI, carotid pulse wave velocity (PWV), and augmentation index (AI).

**Table 2:** Carotid quality arterial stiffness parameters in SLE patients (group A) and controls (group B)

Variable	Group A, median (range)	Group B, median (range)	*z*	*p*
Carotid distension (μm)	300 (86 to 640)	528 (126 to 833)	− 4.427	< 0.001
Compliance coefficient (m^2^/kpa)	0.75 (0.01 to 1.74)	1.31 (0.03 to 2.75)	− 4.805	< 0.001
Distensibility coefficient (1/kpa)	0.02 (0.01 to 0.06)	0.03 (0.01 to 0.08)	− 5.594	< 0.001
PWV (m/s)	7.7 (3.9 to 12)	5.18 (3.8 to 6.8)	− 6.981	< 0.001
SI	8.8 (3.1 to 19)	5.05 (2.77 to 8.29)	− 6.38	< 0.001
AI (%)	4.7 (− 1.6 to 36.0)	0.52 (− 6.7 to 18)	− 3.478	0.001

*PWV* pulse wave velocity, *SI* stiffness index, *AI* augmentation index

For each patient/control, we analyzed the minimal (of both sides) distension, distensibility coefficient, and compliance coefficient and the maximal (of both sides) SI, PWV, and AI.

### Carotid-femoral pulse wave velocity (cf-PWV)

SLE patients (group A) had a higher median cf-PWV of 8.1 m/s (6–14.7), compared to 5.7 m/s (4.7–8.6) in controls (group B) (*z* = − 8.193, *p* ˂ 0.001).

### Comparison between SLE patients and controls after adjusting for hypertension and diabetes mellitus

Since a significant proportion of SLE patients had hypertension and/or DM (46% and 16%, respectively), which are known to influence arterial stiffness parameters and IMT, we repeated our statistical analysis after adjusting for these two factors. The results are shown in Table 3. Linear regression analysis yielded a statistically significant difference between both groups for all parameters (*p* = 0.014 for maximum carotid IMT and < 0.001 for remaining parameters), with the exception of the maximum carotid augmentation index (*p* = 0.184).

**Table 3:** Linear regression analysis to predict arterial stiffness parameters and carotid IMT according to SLE patient/control categorization, after adjusting for diabetes mellitus and hypertension

Variable	B	95% CI	*p*
Aortic strain (%)	− 8.9	− 11.5 to − 6.2	< 0.001
Aortic distensibility	− 5.8	− 7.6 to − 4	< 0.001
Aortic stiffness index	4.9	2.9 to 6.7	< 0.001
FMD (%)	− 10.1	− 12.6 to − 7.6	< 0.001
Carotid IMT (max)	0.07	0.02 to 0.13	0.014
Carotid distension (min)	− 170.4	− 246 to − 94	< 0.001
Carotid CC (min)	− 0.45	− 0.68 to − .022	< 0.001
Carotid DC (min)	− 0.01	− 0.02 to − 0.01	< 0.001
Carotid B-stiffness index (max)	4.5	2.9 to 6.1	< 0.001
Carotid AI (max)	2.3	− 1.1 to 5.6	0.184
Carotid PWV (max)	2.1	1.5 to 2.8	< 0.001
Carotid-femoral PWV (max; corrected)	2.0	1.5 to 2.5	< 0.001

*FMD* flow-mediated dilatation, *IMT* intima media thickness, *CC* compliance coefficient, *DC* distensibility coefficient, *AI* augmentation index, *PWV* pulse wave velocity

### Predictors of arterial stiffness parameters in SLE patients

There was no statistically significant association between stiffness parameters and all tested variables, with the exception of the following:

- Carotid AI (max) and FMD were significantly associated with hypertension, with hypertensive patients having higher median AI compared to non-hypertensives (*z* = − 2.749 and − 2.298, *p* = 0.006 and 0.022, respectively).
- There was a positive association between SLE duration and each of carotid PWV (max), carotid B-stiffness index (max), and cf-PWV (max, corrected) (*r* = 0.363, 0.361, and 0.302, respectively; *p* = 0.01, 0.011, and 0.033, respectively).

### Predictors of carotid IMT

Univariate significant variables included DM (*z* = − 2.317, *p* = 0.021), non-HDL-C level (*r* = 0.366, *p* = 0.009), and SLE duration (*r* = 0.43, *p* = 0.002).

SLE duration was the only significant predictor of IMT by multivariable regression analysis (*R* = 0.493, adjusted *R*^2^ = 0.228, *p* < 0.001).

**Fig. 3:**
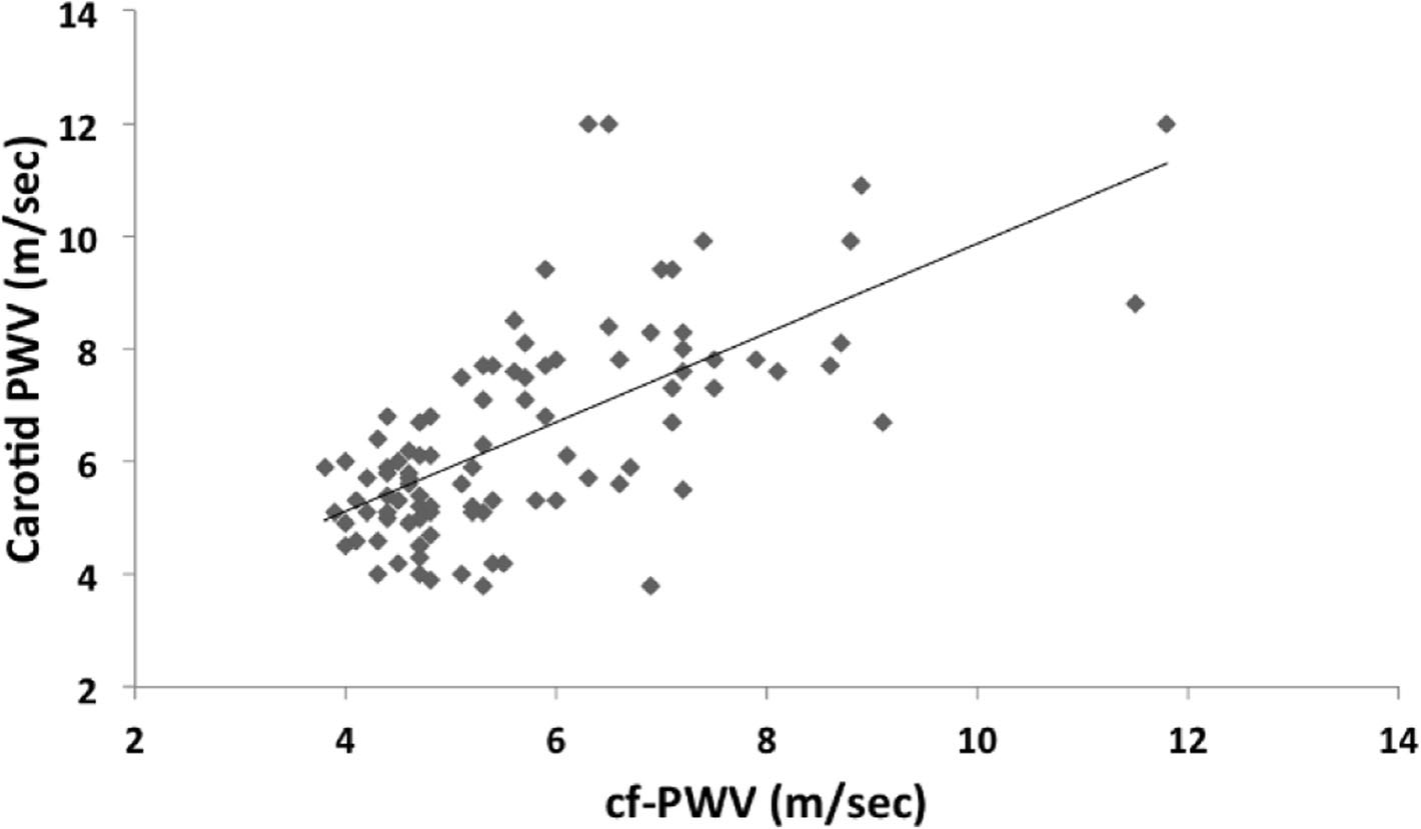
Correlation between cf-PWV and carotid PWV in SLE patients and controls

**Fig. 4:**
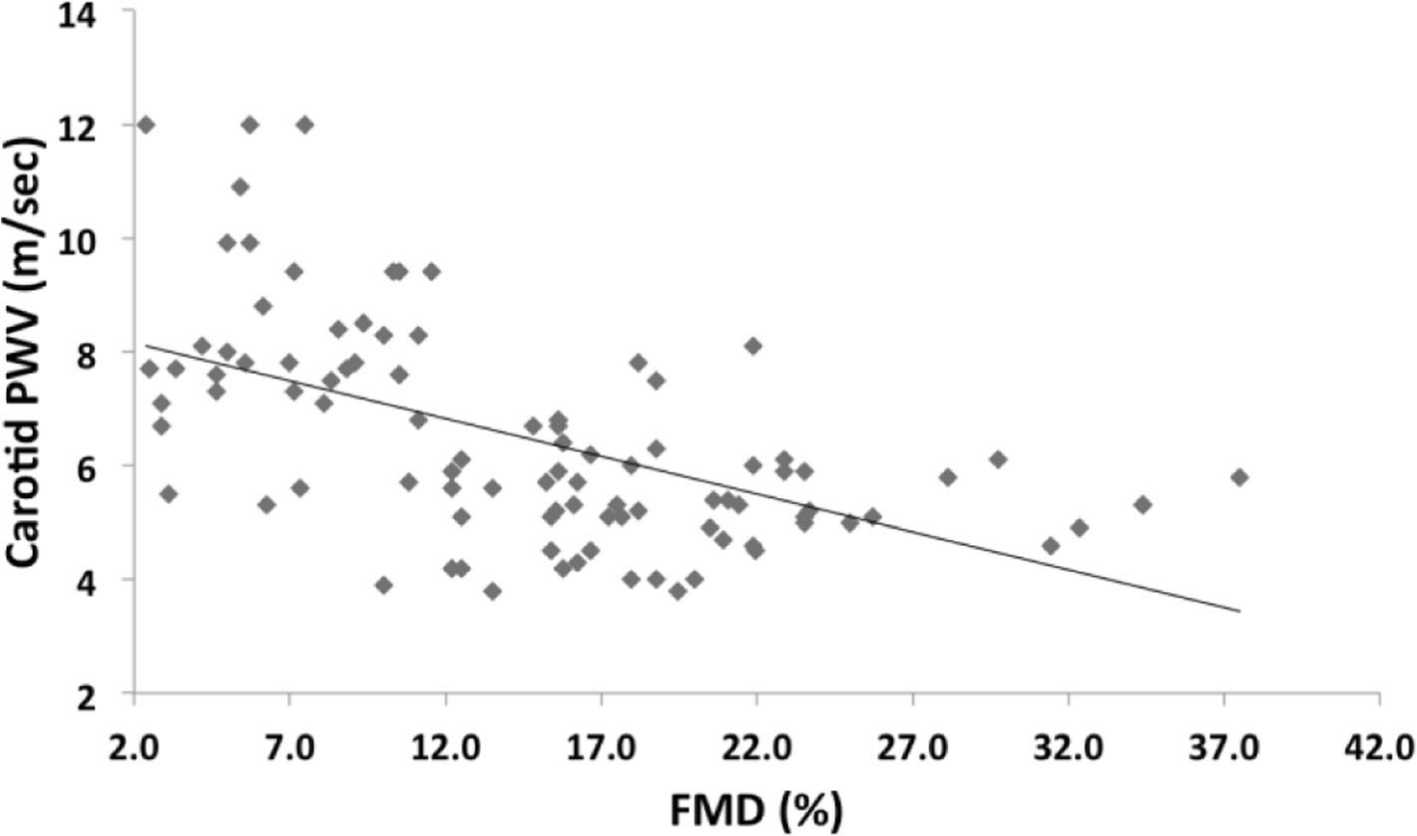
Correlation between FMD and carotid PWV in SLE patients and controls

There was a moderate association between cf-PWV and each of aortic distensibility, aortic SI, carotid PWV (Fig. 3), and carotid SI (*r* = ± 0.55–0.69, *p* < 0.001). Moreover, there was a strong association between cf-PWV and FMD (*r* = − 0.733, *p <* 0.001). FMD correlated moderately with each of aortic distensibility, aortic SI, carotid PWV (Fig. 4), and carotid SI (*r* = ± 0.5–0.65, *p* < 0.001).

### Reliability analysis for measurements of carotid stiffness and cf-PWV

We performed inter-observer variability in a random sample of 5 SLE patients (group A) and 5 controls (group B) for measurements of carotid stiffness and cf-PWV. There was good inter-observer variability regarding measurements of carotid distension, compliance coefficient, SI, carotid PWV, and cf-PWV (intra-class correlation coefficient = 0.83–0.97, *p* ≤ 0.001). On the other hand, there was poor inter-observer variability regarding measurements of carotid distension coefficient and AI (intra-class correlation coefficient = − 0.53 and 0, *p* = 0.95 and 0, 5 respectively).

## Discussion

The majority of SLE patients in the current study were premenopausal females which was consistent with previous studies [22, 23]. Arterial stiffness was proven to predict cardiovascular events and risk beyond the classic risk factors and may be responsible for premature atherosclerosis in SLE. The current study showed that SLE patients had increased arterial stiffness compared with control subjects using a variety of non-invasive modalities.

Using transthoracic echocardiographic M-mode analysis, we demonstrated that SLE patients have significantly increased aortic stiffness compared to controls. SLE patients had significantly higher aortic stiffness index (SI) and lower strain and distensibility. In a study by Roldan et al. [24], 50 patients with SLE (94% women, with a mean age of 38 ± 12 years) and 22 age and gender-matched healthy controls underwent multiplane transesophageal echocardiography showed increased aortic stiffness of the proximal, mid, and distal descending thoracic aorta in SLE patients. In our study, we used transthoracic not transesophageal echocardiography, and therefore, this simple non-invasive tool can be easily integrated into the routine cardiovascular assessment of SLE patients.

Carotid-femoral pulse wave velocity (cf-PWV) is a useful measure of central arterial stiffness and is generally accepted as the simplest, non-invasive, robust, and reproducible method to determine arterial stiffness and may independently predict future CV events and all-cause mortality [25]. There was statistically significant difference between the median cf-PWV of our SLE patients and controls (8.1 m/s vs. 5.7 m/s, *p* ˂ 0.001). In agreement with our findings, Jayapal et al. conducted a study to compare the arterial stiffness among 53 patients with SLE and 53 non-SLE controls; the brachial PWV, the arterial stiffness index, the carotid femoral PWV, and the augmentation index of the SLE patients were significantly higher than that of non-SLE patients (*p* < 0.05) [23]. Also, El Gamal et al. demonstrated that patients with active SLE had significantly higher PWV values than controls (*p* < 0.05), while no difference was found between patients with inactive SLE and controls [26].

Flow-mediated dilatation (FMD) of the brachial artery is the most widely used technique to assess endothelial dysfunction in the macrocirculation [27]. Moreover, endothelial dysfunction has also been found in patients with systemic vasculitis and has been reversed by administration of immunosuppressive therapy [28]. Our study showed that SLE patients have significantly impaired FMD compared to controls. These findings were in agreement with Lima et al. [29] and Kiss et al. [30]. Both reported significantly impaired FMD in SLE patients.

Mendoza-Pinto et al. recently published a large systematic review and meta-analysis of endothelial dysfunction and arterial stiffness in patients with systemic lupus erythematosus including 49 studies. FMD data from 18 studies included 943 SLE subjects and 644 unaffected controls. FMD in SLE subjects was decreased by 4.3% (95% CI − 6.13%, − 2.47%): *p* < 0.001) compared to control groups. Also, they found a significantly increased arterial stiffness between SLE patients and controls according to overall PWV (mean difference = 1.12 m/s; 95% CI 0.72–1.52; *p* < 0.001). Augmentation index was also increased in SLE patients compared with healthy controls (mean difference = 4.55%; 95% CI 1.48–7.63; *p* = 0.003) [31]. Our results were in agreement with this recent meta-analysis, and moreover, we demonstrated a strong association between cf-PWV and FMD (*r* = − 0.733, *p <* 0.001).

SLE-related risk factors such as higher organ damage, activity indices, longer duration of disease, and raised inflammatory biochemical markers were associated with increased arterial stiffness in SLE patients [32]. Cypiene et al. reported strong and significant association between FMD and SLE disease duration [33]. In our study, there was a significant positive correlation between SLE duration and arterial stiffness manifested by increased carotid pulse wave velocity (PWV), carotid B-stiffness index, and carotid-femoral PWV (cf-PWV). Moreover, SLE duration was the only significant predictor of IMT by multivariable regression analysis (*R* = 0.493, adjusted *R*^2^ = 0.228, *p* < 0.001).

Radiofrequency quality arterial stiffness (RF-QAS), an ultrasound method for the assessment of carotid arterial stiffness (CAS), can track the carotid artery wall and measure the change in vessel diameter automatically during cardiac cycles in real-time. CAS is becoming a valuable indicator of future cerebrovascular and cardiovascular events [34]. This method was previously studied in hypertension [35] and renal disease [36]. To the best of our knowledge, our study is the first to apply QAS measures in SLE patients. There was statistically significant difference between our SLE patients and controls regarding all QAS parameters. Compared to controls, SLE patients had significantly lower median carotid distension, distensibility coefficient, and compliance coefficient, with higher median carotid SI, carotid pulse wave velocity, and augmentation index.

Finally, the current study pointed out that increased arterial stiffness and impaired flow-mediated dilatation in SLE patients compared to healthy controls was independent of diabetes mellitus and hypertension. These findings were also highlighted in a comprehensive update of cardiovascular disease in systemic lupus erythematosus by Giannelou and Mavragani [37] in which they reported that traditional CV factors such as smoking, dyslipidemia, diabetes mellitus, hypertension, and central obesity; despite being prevalent in lupus patients, they do not fully explain the high rates of ischemic events so far reported, implying that other factors inherent to disease itself could account for the enhanced risk.

## Limitations

This study had some limitations being a cross-sectional one with no follow-up to determine whether the stiffness parameters would change with fluctuations in SLE disease activity.

## Conclusion

Our study demonstrated that SLE could be an independent cardiovascular risk factor, with impaired endothelial function and increased arterial stiffness in SLE patients compared to healthy controls. Arterial stiffness parameters were positively associated with SLE duration. These findings emphasize the need for early diagnosis of SLE and aggressive risk factor modification for primary and secondary prevention of arterial stiffness and atherosclerosis. These interventions may have the potential to decrease the prevalence and incidence of cardiac and cerebrovascular morbidity and mortality in SLE patients.

## Data Availability

The datasets used and/or analyzed during the current study are available from the corresponding author on reasonable request.

## Abbreviations

ACR: American College of Rheumatology
AI: Augmentation index
CAD: Coronary artery disease
cf-PWV: Carotid-femoral pulse wave velocity
FMD: Flow-mediated dilation
IMD: Intima media thickness
PWV: Pulse wave velocity
QAS: Quality arterial stiffness
SLE: Systemic lupus erythematosus
SLICC: Systemic Lupus International Collaborating Clinics
TTE: Transthoracic echocardiography
